# Promyelocytic leukemia zinc finger is involved in the formation of deep layer cortical neurons

**DOI:** 10.1186/s12929-019-0519-8

**Published:** 2019-04-26

**Authors:** Hsin-Chuan Lin, Yung-Hao Ching, Chi-Chen Huang, Ping-Chieh Pao, Yi-Hua Lee, Wen-Chang Chang, Tzu-Jen Kao, Yi-Chao Lee

**Affiliations:** 1 Graduate Institute of Medical Sciences, College of Medicine, Taipei Medical University, Taipei, Taiwan; 2 Department of Molecular Biology and Human Genetics, Tzu Chi University, Hualien, Taiwan; 3 PhD Program for Neural Regenerative Medicine, College of Medical Science and Technology, Taipei Medical University, Taipei, Taiwan; 4 Center for Neurotrauma and Neuroregeneration, Taipei Medical University, Taipei, Taiwan; 5 Department of Pharmacology, College of Medicine, National Cheng Kung University, Tainan, Taiwan; 6 Ph.D Program in Biotechnology Research and Development, College of Pharmacy, Taipei Medical University, Taipei, Taiwan

**Keywords:** Promyelocytic leukemia zinc finger, Plzf, *luxoid* mice, Cortical neuron

## Abstract

**Background:**

Promyelocytic leukemia zinc finger (Plzf), a transcriptional regulator involved in a lot of important biological processes during development, has been implied to maintain neural stem cells and inhibit their differentiation into neurons. However, the effects of Plzf on brain structures and functions are still not clarified.

**Results:**

We showed that Plzf expression was detected as early as embryonic day (E) 9.5 in Pax6^+^ cells in the mouse brain, and was completely disappeared in telencephalon before the initiation of cortical neurogenesis. Loss of Plzf resulted in a smaller cerebral cortex with a decrease in the number of Tbr1^+^ deep layer neurons due to a decrease of mitotic cell number in the ventricular zone of forebrain at early developmental stage. Microarray, qRT-PCR, and flow cytometry analysis identified dysregulation of *Mash1* proneural gene expression. We also observed an impairment of recognition memory in *Plzf*-deficient mice.

**Conclusions:**

Plzf is expressed at early stages of brain development and involved in the formation of deep layer cortical neurons. Loss of Plzf results in dysregulation of Mash1, microcephaly with reduced numbers of early-born neurons, and impairment of recognition memory.

**Electronic supplementary material:**

The online version of this article (10.1186/s12929-019-0519-8) contains supplementary material, which is available to authorized users.

## Background

Promyelocytic leukemia zinc finger (*PLZF*, also known as *ZBTB16*, *ZNF145*) is a *kruppel*-like C2H2 zinc finger gene containing nine zinc fingers in the C-terminus and a BTB/POZ domain in the N-terminus [1] and has been shown to mediate a lot of important biological processes, especially hematopoiesis, osteogenesis, and spermatogenesis [2–5]. The patient with biallelic loss of *PLZF* function exhibits abnormality in bone development, genital hypoplasia, and microcephaly with a severe mental retardation, implying the involvement of *PLZF* in brain development [6, 7]. Actually, Plzf expression was observed in the anterior neuroepithelium at early stage (E7.5), which later extends to the entire neuroectoderm until stage E10 [8]. In zebrafish, Plzf can block neuronal differentiation by inhibiting the expression of proneural gene Ngn1 [9]. A study also showed that Plzf maintains neural progenitors in the spinal cord of chick and mouse by up-regulating FGFR3 expression and through STAT3 activation [10]. Although, these results suggest that PLZF is important for the development of central nervous system, the effects of Plzf on brain structures and functions are still not clarified.

The development of cerebral cortex is an important and complicated process which involves in neural stem cells (NSCs) proliferation, differentiation, cell fate determination, and cell migration [11–13]. During brain development, NSCs can either divide symmetrically to expand the cell pool through the process called self-renewal, or undergo the neurogenesis process to divide asymmetrically and generate neural progenitors that are subsequently differentiated into neurons [14]. The proper balance between NSCs self-renewal and neurogenesis is important to ensure appropriate numbers of neurons being generated. Indeed, defects affecting this balance have been suggested to induce brain malformations [15–17].

To better understand the effect of Plzf in mammalian brain development, we first examined the cell type-restricted expression of Plzf during mouse brain development. We then characterized the developmental brain abnormality and the behavioral phenotypes of the *Plzf*-deficient mice. In addition, we also performed microarray to examine the differences in gene expression profiles of developmental brain between wild-type and *Plzf*-deficient mice. Our results revealed that Plzf is expressed at early stages of brain development and involved in the formation of deep layer cortical neurons.

## Methods

### Animals

The animal protocols used in this study complied with the guidelines of the Taipei Medical University Institutional Animal Care and Use Committee (IACUC). All experiments were performed in accordance with the US Public Health Service Policy on Humane Care and Use of Laboratory Animals. All animals were housed in an air-conditioned vivarium with free access to food and water and a 10/14 h light/dark cycle.

### Antibodies

The following antibodies were used, including Satb2, Ctip2, Tbr1, and Mash1 (Abcam, Cambridge, CB, UK), Plzf and PHH3 (Santa Cruz, Santa Cruz, CA, USA), Pax6 (Merck Millipore, Darmstadt, Hessen, Germany), β-actin (Sigma-Aldrich, Louis, MO, USA).

### Magnetic resonance imaging

Mouse brains at postnatal day (P) 0 were fixed with 4% paraformaldehyde for 48 h and embedded in 1.5% agarose. All images were acquired using Avance spectrometer (Bruker, Karlsruhe, Baden-Württemberg, Germany) with 9.4 T WB 8.9-cm bore vertical system (Oxford Instrument, Oxford, UK). A 3D fast spin-echo (FSE) T2WI sequence was used to acquire anatomical images for volumetric analysis with the following parameters: a repetition time (TR) of 10,000 ms, an effective echo time (TE) of 91 ms, a RARE factor of 8, a field-of-view (FOV) of 0.9 cm, an acquisition matrix of 256 × 128 × 30 (zero-padded to 512 × 512 × 30), a resolution of 0.0039 × 0.0078 × 0.0073 cm/pixel. Brain volumes were measured using the manual tracing utility in Avizo software (Visualization Sciences Group, Merignac, Aquitaine, France).

### Behavioral studies

#### Open field test

The open field activity was assessed in a chamber (57.4 cm × 59 cm × 95 high cm). Mice were individually placed in the middle of the arena and allowed to explore for 10 min. The moving trace of each mouse was recorded by a video camera and analyzed by the EthoVision XT software (Noldus Information Technology, Leesburg, VA, USA).

#### Novel object recognition (NOR) test

NOR test was assessed in a chamber (57.4 cm × 59 cm × 95 high cm). The test consists of three sessions: habituation, familiarization (training), and test session. In the habituation session, each mouse was allowed to freely explore the arena in the absence of objects for 10 min. The mice were then removed from the arena and placed in its holding cage. One day after the habituation session, mice were subjected to a single familiarization session of 10 min. During the familiarization session, each mouse was placed in the same arena containing two identical sample objects (A + A). One day after the familiarization session, mice were subjected to a single test session of 10 min: the animal was placed in the arena with two objects, including one sample (A) and one novel object (B). During both the familiarization and test sessions, the time the mice spent exploring each object was recorded.

### Immunohistochemistry and immunofluorescence

For preparing embryonic sections, the embryos were fixed with 4% paraformaldehyde (PFA) in phosphate buffered saline (PBS), pH 7.4 at 4 °C for overnight. Fixed embryos were immersed in 30% sucrose in PBS and then frozen in OCT and cryosectioned to 25 μm sections using the Leica CM1950 freezing microtome (Leica Biosystems, Wetzlar, Hesse, Germany). For the preparation of postnatal brain sections, brains were fixed with 4% PFA in PBS, pH 7.4 at 4 °C for 48 h. Fixed brains were then dehydrated with 75% alcohol prior to paraffin embedding. 3 μm thick sections were obtained using Sakura IVS-410 microtome (Sakura Seiki Co, Tokyo, Japan). All the paraffin sections were dewaxed and rehydrated prior to the staining procedures. For both immunohistochemistry and immunofluorescence staining, tissue sections were permeabilized and blocked with blocking buffer (0.2% Triton X-100, 10% goat serum in PBS) at room temperature for 1 h. For immunofluorescence, tissue sections were incubated with primary antibody solution overnight at 4 °C. After extensive washes with PBS, the tissue sections were incubated with the corresponding secondary antibodies at room temperature for 1 h. For immunohistochemistry, the tissue sections were incubated with primary antibodies overnight at 4 °C. After extensive washes with PBS, the tissue sections were incubated with biotinylated secondary antibodies. The colorimetric detection of primary antibodies was developed using the Vectastain ABC Kit (Vector laboratories, Burlingame, California, USA), followed by exposure to peroxidase DAB substrate (Vector laboratories). Cell nuclei were stained with hematoxylin (Leica) or Hoechst 33342 (Sigma-Aldrich) and slides were mounted with anti-fading solution (SouthernBiotech, Birmingham, AL, USA).

### Image analysis

Tissue sections were observed and photographed by Leica STP6000 fluorescent microscope (Leica Biosystems) and scanned by TissueGnostics TissueFAXS & HistoFAXS (TissueGnostics Gmbh, Vienna, Austria). Images were overlaid by MetaMorph software (Universal Imaging Corporation, Philadelphia, PA, USA**),** and the brightness and contrast of images were adjusted by Photoshop (Adobe, Camarillo CA, USA) as necessary. With regard to the measurement of cerebral cortex area, comparable 3 μm-thick sagittal sections of P7 mice brain were stained by hematoxylin and eosin (H&E). The area of the cortical layers was measured by HistoQuest software (TissueGnostics GmbH). The numbers of Tbr1-, Satb2-, and Ctip2-positive cells was quantified by TissueQuest software (TissueGnostics GmbH). The number of PHH3-positive cells was quantified in 120 μm-wide sampling boxes along the ventricular zone of the telencephalon. The quantification of each experimental group was repeated on two non-adjacent sections for each embryo.

### Western blot analysis

Embryos were homogenized by radioimmunoprecipitation assay (RIPA) buffer (150 mM NaCl, 1% NP40, 0.5% Na-deoxycholate, 0.1% sodium dodecylsulfate (SDS), and 50 mM Tris-HCl; pH 8.0) with the addition of a protease inhibitor cocktail used for homogenization. For embryos younger than E11.5, three heads were pooled together as one biological sample. After lysed, the cell debris were removed with centrifugation fall at 12,000 rpm for 5 min at 4 °C, and the supernatants were stored at − 80 °C. Protein concentration was determined using the Bio-Rad dye-binding method with bovine serum albumin (BSA) as the standard. Equal amounts of samples were separated by 8–16% gradient polyacrylamide gel electrophoresis (PAGE). The resolved proteins were then electroblotted onto Immobilon polyvinylidene difluoride (PVDF) membranes (PerkinElmer, Waltham, MA, USA), which was followed by blocking with 5% low-fat milk. Blotted membranes were then incubated with selected primary antibodies overnight at 4 °C. After extensive washes, membranes were incubated with the corresponding secondary antibodies at room temperature for 1 h. After washing, the membranes were processed for visualization using an enhanced chemiluminescence (ECL) system (PerkinElmer, Waltham, MA, USA). Membranes were then exposed to x-ray film to obtain the fluorographic images and quantified by ImageJ (NIH, Bethesda, MD, USA). Values for each lane were normalized to β-actin.

### Microarray and gene ontology (GO) analysis

RNA was extracted from forebrain and midbrain regions of mouse E10.5 embryos with TRIzol reagent (Invitrogen). 0.2 μg of total RNA was amplified by a Low Input Quick-Amp Labeling kit (Agilent Technologies, USA) and labeled with Cy3 or Cy5 (CyDye) (Agilent Technologies, California, USA) during the in vitro transcription process. 0.825 μg of Cy-labeled cRNA was fragmented to an average size of about 50–100 nucleotides by incubation with fragmentation buffer at 60 °C for 30 min. Correspondingly fragmented labeled cRNA was then pooled and hybridized to an Agilent SurePrint G3 Mouse Gene Exp v2 Array Kit (Agilent Technologies) at 65 °C for 17 h. After washing and drying by nitrogen gun blowing, microarrays were scanned with an Agilent microarray scanner (Agilent Technologies) at 535 nm for Cy3 and 625 nm for Cy5. Scanned images were analyzed by Feature extraction 10.7.3.1 software (Agilent Technologies), and image analysis and normalization software was used to quantify signal and background intensity for each feature, substantially normalized the data by rank-consistency-filtering LOWESS method. The spots with the processed signal higher than 200 and the signal-to-noise ratio (SNR) greater or equal to 5 were considered positive signals. The raw data were available at http://www.ncbi.nlm.nih.gov/geo/ (accession numbers: GSM3273361~3,273,362, GSE117197). The genes with greater or less than 1.3-fold changes in expression between wild-type and *Plzf-deficient* mice were selected for further analysis. GO analysis were performed using ToppFun (https://toppgene.cchmc.org/) and Ingenuity Pathways Analysis (IPA) (Ingenuity, Redwood City, CA, USA).

### Quantitative RT–PCR

Reverse transcription (RT) was performed with 1.5 μg of total RNA using M-MLV reverse transcriptase (Invitrogen). A real-time qPCR was performed using the iTaq Universal SYBR Green Supermix (Biosystem Applications, Foster City, CA, USA) with the following conditions: step1, 95 °C for 30 s; step2 (repeated for 40 cycles), 95 °C for 5 s, 60 °C for 30 s. Real-time fluorescence monitoring and a melting-curve analysis were performed by StepOnePlus Real-Time PCR System according to the manufacturer’s recommendations (Life Technologies, Carlsbad, CA, USA). Negative controls containing no complementary (c)DNA template were included in each experiment. A melting curve was created at the end of the PCR cycle to confirm that a single product had been amplified. Data were analyzed by StepOne Software version 2.2.2 (Life Technologies) to determine the threshold cycle (*Cp*) above the background for each reaction. The relative transcript amount of the target gene, calculated using standard curves of serial cDNA dilutions, was normalized to that of *Gapdh* of the same cDNA. Primers used in PCR assays were as follows: *Mash1,* forward primer, 5′- TTGAACTCTATGGCGGGTTC-3′, reverse primer, 5′-GGTTGGCTGTCTGGTTTGTT-3′; *Gapdh*, forward primer, 5′- TGACATCAAGAAGGTGGTGAAG-3′, reverse primer, 5′- AGAGTGGGAGTTGCTGTTGAAG-3′.

### Flow cytometry

Forebrain and midbrain tissues were dissected from E10.5 mouse embryos and dissociated by 0.2% Trypsin/EDTA at 37 °C for 5 min, and then filtered through a 70-μm nylon mesh filter (Corning life science, Corning, New York, USA). The isolated cells were fixed by 75% methanol. For cell cycle analysis, the cells were washed once with ice-cold PBS and labeled with propidium iodide (PI) (Sigma) at 25 °C for 1 h. Five thousand events without cell debrides were then analyzed using the Guava EasyCyte system and the InCyte software (Millipore). For analysis of Mash1-expressing cells, the cells were washed once with PBS and incubated with anti-Mash1 antibody (Abcam) with PI solution in blocking buffer (10% normal goat serum, 0.2% RNaseA, and 0.1% Triton-100X in PBS) for 1 h at 25 °C. The cells were washed and incubated with Alexa Fluor 488 anti-mouse IgG (Life Technologies) for 1 h at 25 °C. After washed with ice-cold PBS to remove the unconjugated antibodies, the cells were resuspended in PBS, and 5000 events without cell debrides were analyzed using the Guava EasyCyte system and the InCyte software (Millipore).

## Results

### Plzf is temporarily expressed in Pax6^+^ cells at early stages of brain development

**Fig. 1:**
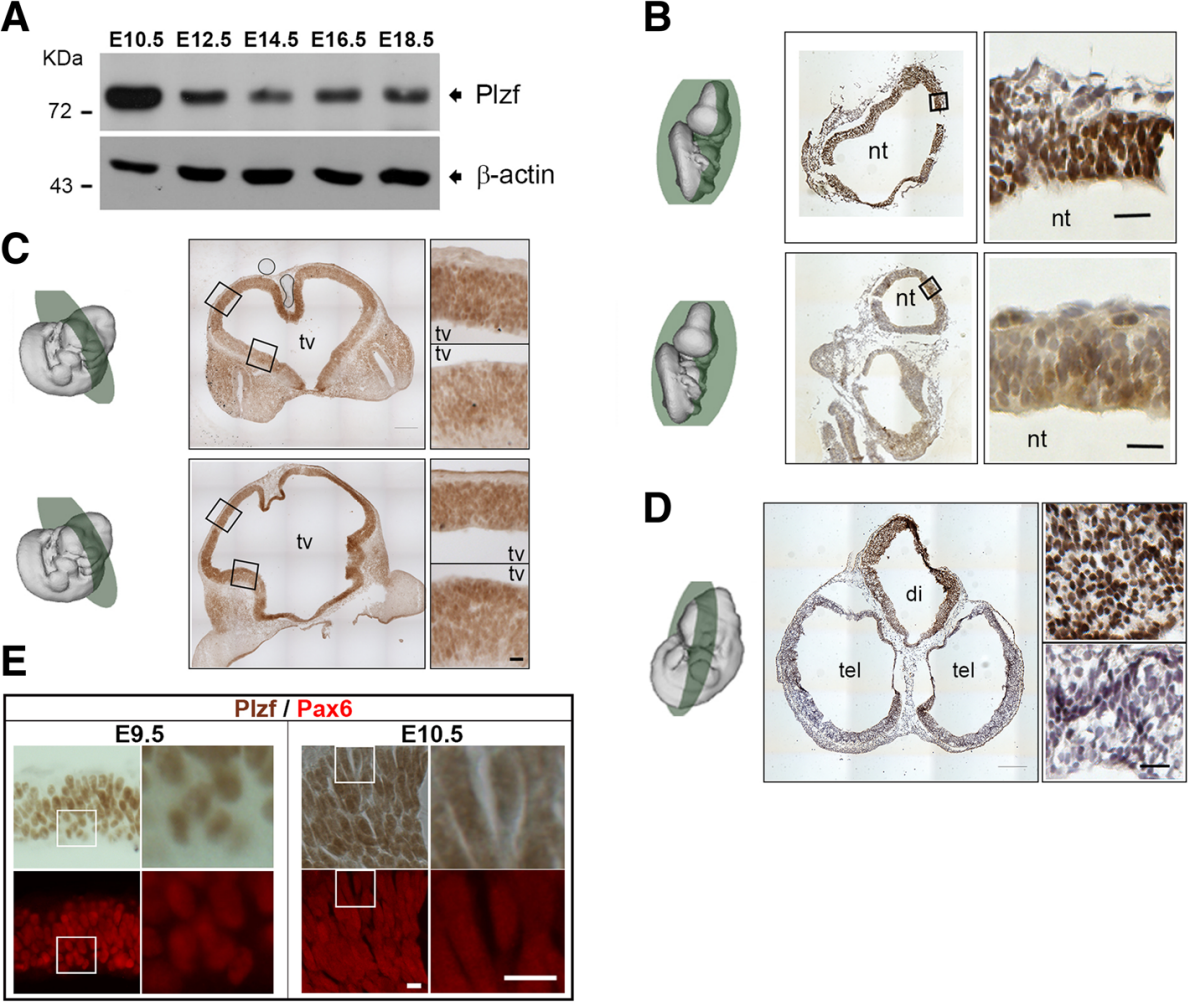
Plzf temporarily expressed in Pax6^+^ cells at early stages of brain development. **a**, Western blot analysis of Plzf expression in whole brain lysates of mouse embryo at different stages. **b-d**, 20 μm frozen sections were prepared from the E9.5 (**b**), E10.5 (**c**), and E11.5 (**d**) embryos and immunohistochemistry for Plzf was performed with DAB (brown) followed by Hematoxylin-eosin (HE) counterstaining. The diagrams of mouse embryo were obtained from e-Mouse Atlas Project (http://www.emouseatlas.org/emap/ema/home.php) and the green circles indicate the approximate plane of the cross section. The higher magnification images of the black boxes were shown in the right panels. Scale bar, 20 μm. **e**, The expressions of Pax6 (red) and Plzf (brown) were examined by immunostaining in the same brain sections. The higher magnification images of the white boxes were shown in the right panels. E, embryonic day; nt, neural tube; tv, third ventricle; tel., telencephalon; di, diencephalon. Scale bar, 10 μm

Previous studies have shown that Plzf is expressed in the anterior neuroepithelium of mouse embryo at E7.5 and spreading to the entire neuroectoderm until E10 [8]. We therefore examined the expression pattern of Plzf by Western blotting and immunostaining in the brains of mouse embryos at different stages. Similar to previous study, we found Plzf was highly expressed in the mouse embryonic brains at E10.5, and the expression was decreased after E12.5 (Fig. 1a). Immunostaining results showed that a high expression level of Plzf in the prosencephalon, which later becomes the forebrain, at E9.5 (Fig. 1b) and E10.5 (Fig. 1c). Further examinations showed that Plzf expression was dramatically decreased in telencephalon at E11.5 (Fig. 1d). We also found that Plzf was expressed in Pax6^+^ cells in prosencephalon at E9.5 and E10.5 (Fig. 1e), implying a role of Plzf on neuroepithelial cells or radial glial cells at early stages of neurogenesis.

### *Plzf*-deficient mice display a decrease in cerebral cortex size and the number of deep-layer cortical neurons

**Fig. 2:**
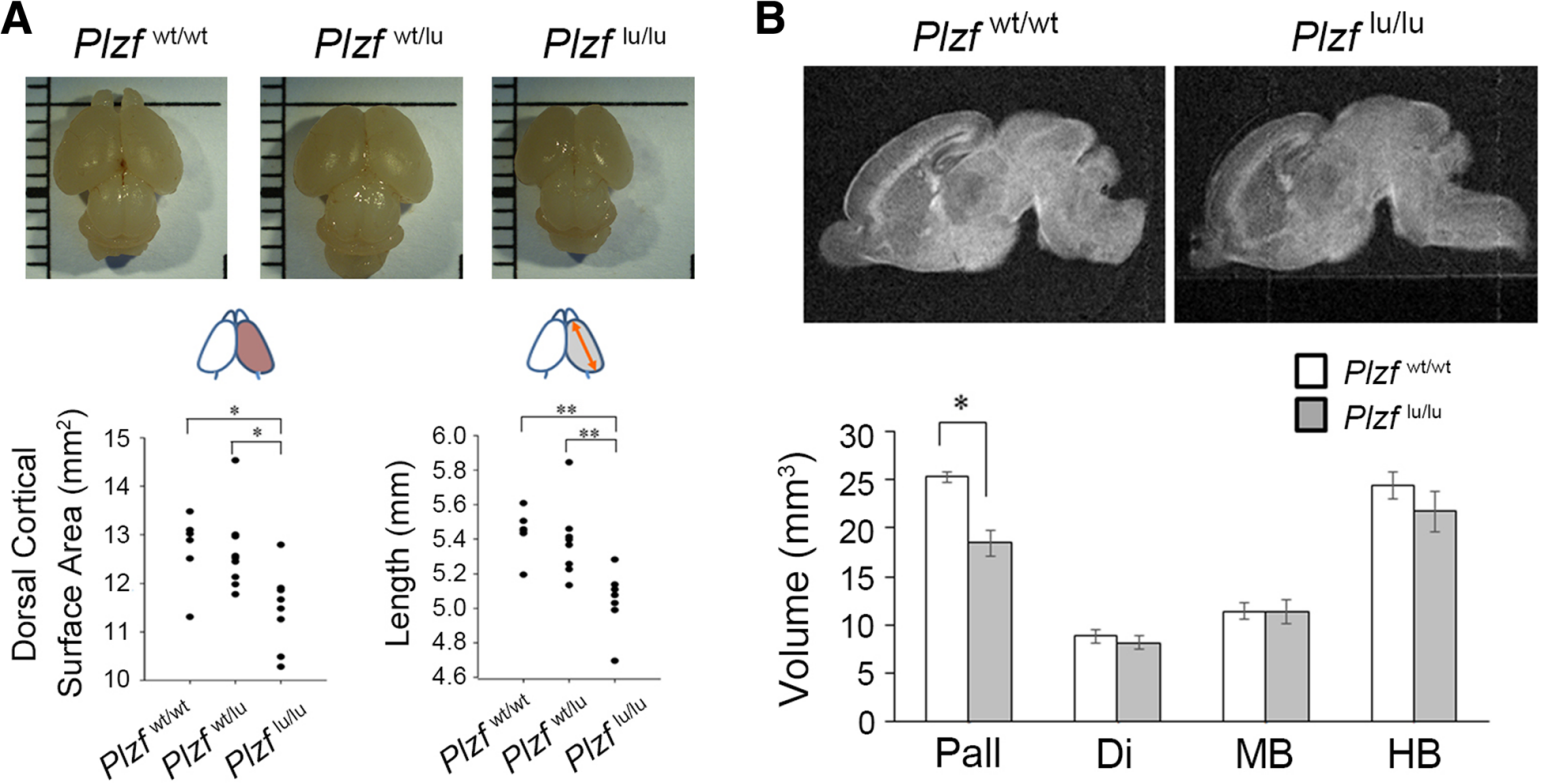
*Plzf*-deficient mice exhibit smaller cerebral cortex. **a**, Dorsal view of whole-mount brains at P0. For the scale size, each of the thin line indicates 1 mm, and the thick line indicates 5 mm. Quantifications of dorsal cortical surface area and cortical length shown in the lower panels. Statistical analysis performed using one-way ANOVA with appropriate post hoc tests: *p < 0.05 and **p < 0.01; *Plzf*^wt/wt^, *n* = 6; *Plzf*^wt/lu^, *n* = 9; *Plzf*^lu/lu^, *n* = 8. **b**, Representative sagittal views of 9.4 T MRI sections of *Plzf*^wt/wt^ and *Plzf*^lu/lu^ mice brain. Quantification of the volume of brain regions shown in the lower panel. Statistics performed with Student’s *t* test: *p < 0.05; *n* = 4 per genotype. Data are represented as mean ± SEM. P, postnatal day; *Plzf*^wt/wt^, wild-type; *Plzf*^wt/lu^, heterozygous; *Plzf*^lu/lu^, homozygous *luxoid* mice; Pall, Pallium; Di, diencephalon; MB, midbrain; HB, hindbrain

To explore the effect of Plzf in mammalian brain development, we examined the possible structural and functional alterations in the brain of *Plzf*-deficient (luxoid; *lu*) mice, *Plzf*-mutant mice which arose spontaneously in 1950’s and was characterized with a single nucleotide change in the first coding exon of *Plzf* gene that resulted a severely truncated protein [3]. We first found that the dorsal cortical surface area and hemisphere length were significantly reduced in *Plzf*-deficient mice (*Plzf*^lu/lu^) at postnatal day (P) 0 when compared to wild type (*Plzf*^wt/wt^) and heterozygous (*Plzf*^wt/lu^) littermates (Fig. 2a). At P0, the average of dorsal cortical area were 12.717 ± 0.338 mm^2^ in *Plzf*^wt/wt^, 12.659 ± 0.288 mm^2^ in *Plzf*^wt/lu^, and 11.467 ± 0.305 mm^2^ in *Plzf*^lu/lu^, respectively, and the average lengths of hemisphere were 5.441 ± 0.061 mm in *Plzf*^wt/wt^, 5.390 ± 0.071 mm in *Plzf*^wt/lu^, and 5.076 ± 0.071 mm in *Plzf*^lu/lu^, respectively (Fig. 2a). Using 3D MRI imaging, we accurately measured the volumes of brain regions at P0 and found that when compared to wild-type littermates, *Plzf*^lu/lu^ mice showed a significantly decrease in the volume of pallium (the cortex and hippocampus) but not in other brain areas (Fig. 2b).

**Fig. 3:**
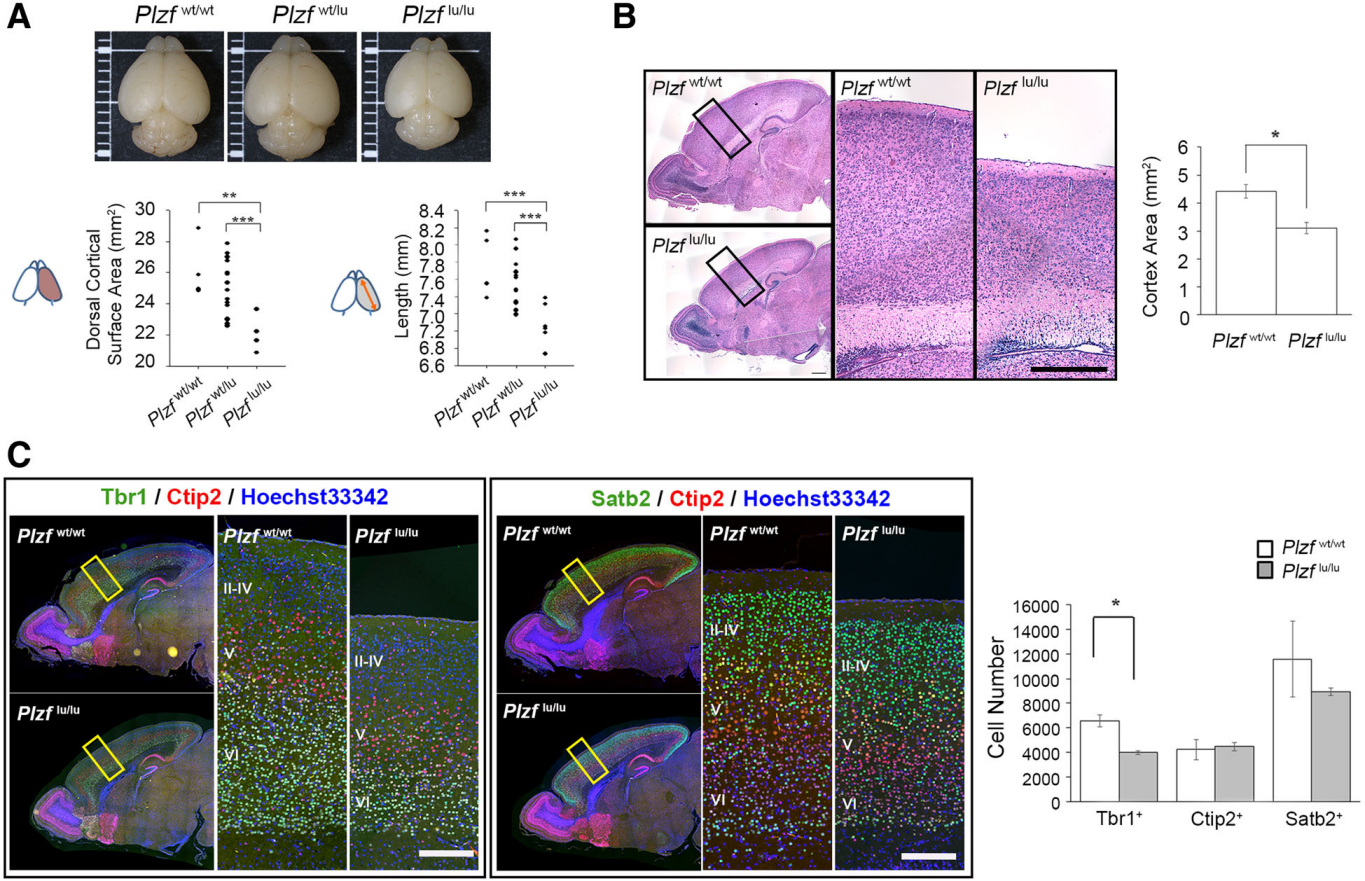
Reduced numbers of neurons in deep-layer cortex of *Plzf*^lu/lu^ mice. **a**, Dorsal view of whole-mount brains at P7. For the scale size, each of the thin lines indicates 1 mm, and the thick line indicates 5 mm. Quantifications of dorsal cortical surface area and cortical length shown in the lower panels. Statistical analysis performed using one-way ANOVA with appropriate post hoc tests: **p < 0.01 and ***p < 0.001. *Plzf*^wt/wt^, *n* = 7; *Plzf*^wt/lu^, *n* = 17; *Plzf*^lu/lu^, *n* = 9. **b**, HE staining of sagittal brain sections, the higher magnification images of the black boxes were shown in the middle and right sides of the panel. Quantification of the area of cerebral cortex from rostral to caudal shown in the right panel. Statistics performed with Student’s *t* test: *p < 0.05; *n* = 3 per genotype. **c**, The left panel showed immunofluorescence images of *Plzf*^wt/wt^ and *Plzf*^lu/lu^ mice brain sections. Tbr1 as layer VI neuron marker (green), Ctip2 as layer V neuron marker (red) and Hoechst33342 for nucleus staining (blue). For the middle panels, Satb2 as layer II-III marker (green). The higher magnification images of the yellow boxes were shown in the middle and right sides of panels. Quantification of the Tbr1^+^, Satb2^+^, and Ctip2^+^ neuron numbers in cerebral cortex shown in the right panel. Statistics performed with Student’s *t* test: *p < 0.05; n = 3 per genotype. Values represent the mean ± SEM. P, postnatal day; *Plzf*^wt/wt^, wild-type; *Plzf*^wt/lu^, heterozygous; *Plzf*^lu/lu^, homozygous; Scale bar, 200 μm

Similar to observations at P0, the dorsal cortical surface area and hemisphere length were also significantly reduced in *Plzf*^lu/lu^ mice at P7 (Fig. 3a). Our data showed that the average of dorsal cortical area at P7 is 25.202 ± 0.748 mm^2^ in *Plzf*^wt/wt^, 24.852 ± 0.434 mm^2^ in *Plzf*^wt/lu^, and 21.747 ± 0.564 mm^2^ in *Plzf*^lu/lu^, respectively, and the average of hemisphere length is 7.619 ± 0.146 mm in *Plzf*^wt/wt^, 7.521 ± 0.068 mm in *Plzf*^wt/lu^, and 6.984 ± 0.103 mm in *Plzf*^lu/lu^, respectively (Fig. 3a). We also evaluated the cerebral cortical area from haematoxylin and eosin (H&E) stained brain sections, and found that the cortical area was significantly decreased in *Plzf*^lu/lu^ mice (Fig. 3b). Our data showed that the *Plzf*^lu/lu^ mice have a thinner cortex. Using immunostaining, we further evaluated the alteration of cortical structure in *Plzf*^lu/lu^ mice labelled with specific cortical layer markers, such as Tbr1 (a marker of cortical deep-layer neurons), Ctip2 (a marker of layer 5 neurons), and Satb2 (a marker of layer 2/3 neurons). Our result showed a significantly decreased number of Tbr1^+^ neurons in layer VI of *Plzf*^lu/lu^ mouse brain cortex (Fig. 3c). On the other hand, the numbers of Ctip2^+^ neurons and Satb2^+^ neurons were not different between *Plzf*^wt/wt^ and *Plzf*^lu/lu^ mice. Our results thus indicate that loss of Plzf results in a decrease in the numbers of early-born neurons, suggesting the requirement of Plzf in early stage of neurogenesis.

### Cell proliferation and gene expression changes in *Plzf-*deficient mice

**Fig. 4:**
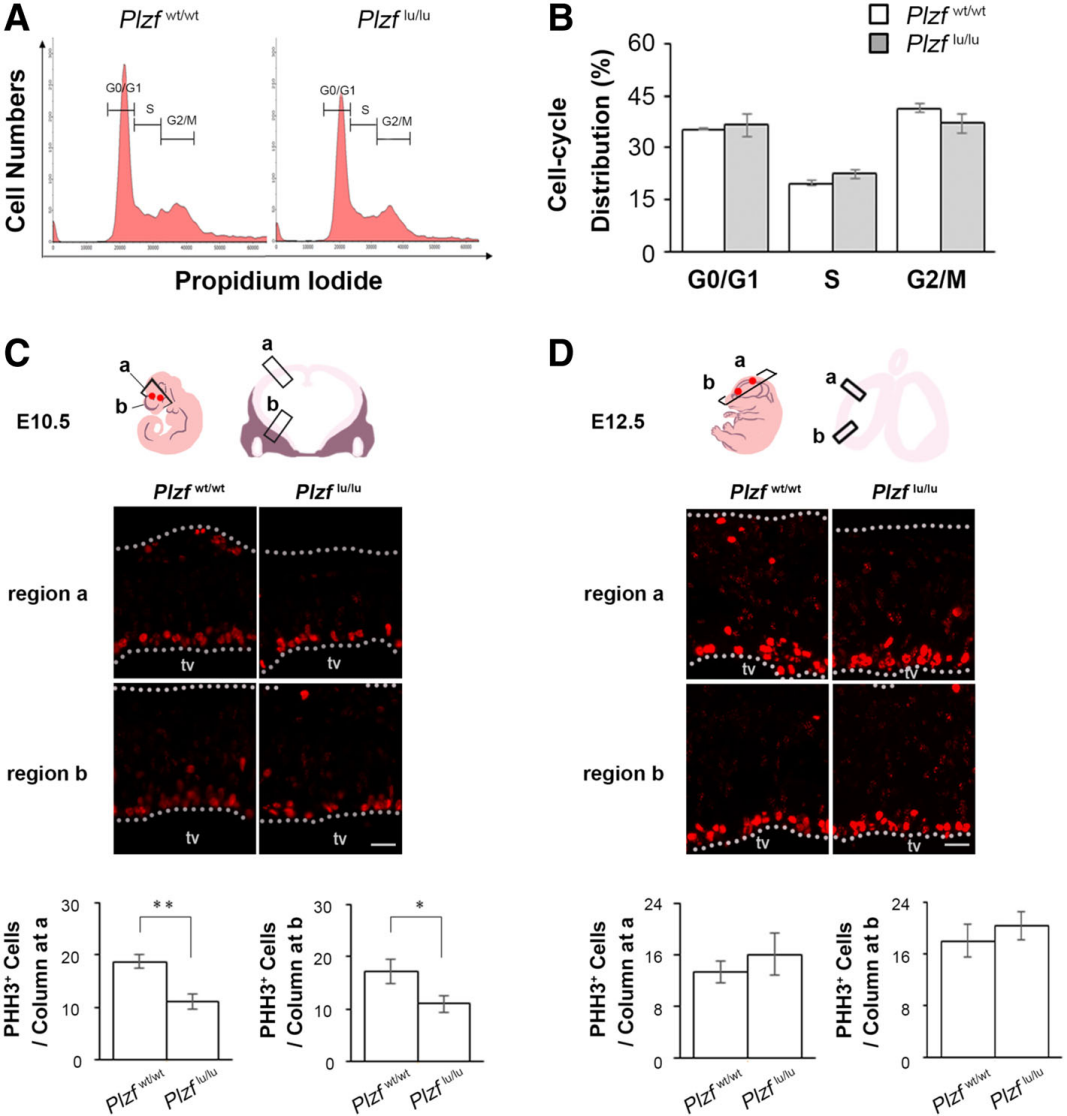
Cell proliferation change at early stage of neurogenesis in *Plzf*^lu/lu^ mice brain. **a,** Representative flow cytometric data showing the cell cycle distribution. X-axis is the intensity of propidium iodide, and Y-axis is the cell numbers. **b**, Quantification of proportions of cells in each phase of the cell cycle. *Plzf*^wt/wt^, *n* = 4; *Plzf*^lu/lu^, *n* = 3. **c** and **d**, E10.5 (***c***) and E12.5 (***d***) brain sections were immunstained by anti-PHH3 antibodies. The PHH3^+^ cells were calculated in the rostral (b) and caudal (a) regions, respectively. The white dashed lines represent the apical and basal borders of cerebral cortex. Quantification of the PHH3^+^ cell numbers in the region shown in the lower panels. n = 4 embryos per genotype at E10.5; *n* = 3 embryos per genotype at E12.5. Statistics were performed with Student’s *t* test. Values represent the mean ± SEM. E, embryonic day; *Plzf*^wt/wt^, wild-type; *Plzf*^lu/lu^, homozygous *luxoid* mice; tv, third ventricle; Scale bar 20 μm

To evaluate the possible mechanism of cortical thinning in *Plzf-*deficient mice, we first analyzed the cell proliferation in the brain of wild type and *Plzf*^lu/lu^ mice at E10.5 by flow cytometry. Although not significant, our result showed that there was a slight decrease in G2/M phase cells in *Plzf*^lu/lu^ mice (Fig. 4a and b). To accurately measure the cell proliferation in the ventricular zone of the prosencephalon, we further analyzed the amount of mitotic cells in the ventricular zone of prosencephalon in wild-type and *Plzf*^lu/lu^ mice by using phospho-histone H3 (PHH3) immunostaining. As shown in Fig. 4c and d, the number of mitotic cell in the ventricular zone of prosencephalon is significantly decreased in *Plzf*^lu/lu^ mice at E10.5 (Fig. 4c) but not at E12.5 (Fig. 4d), indicating cell proliferation change at early stage of neurogenesis may cause the abnormal cortical development in *Plzf*^lu/lu^ mice.

**Fig. 5:**
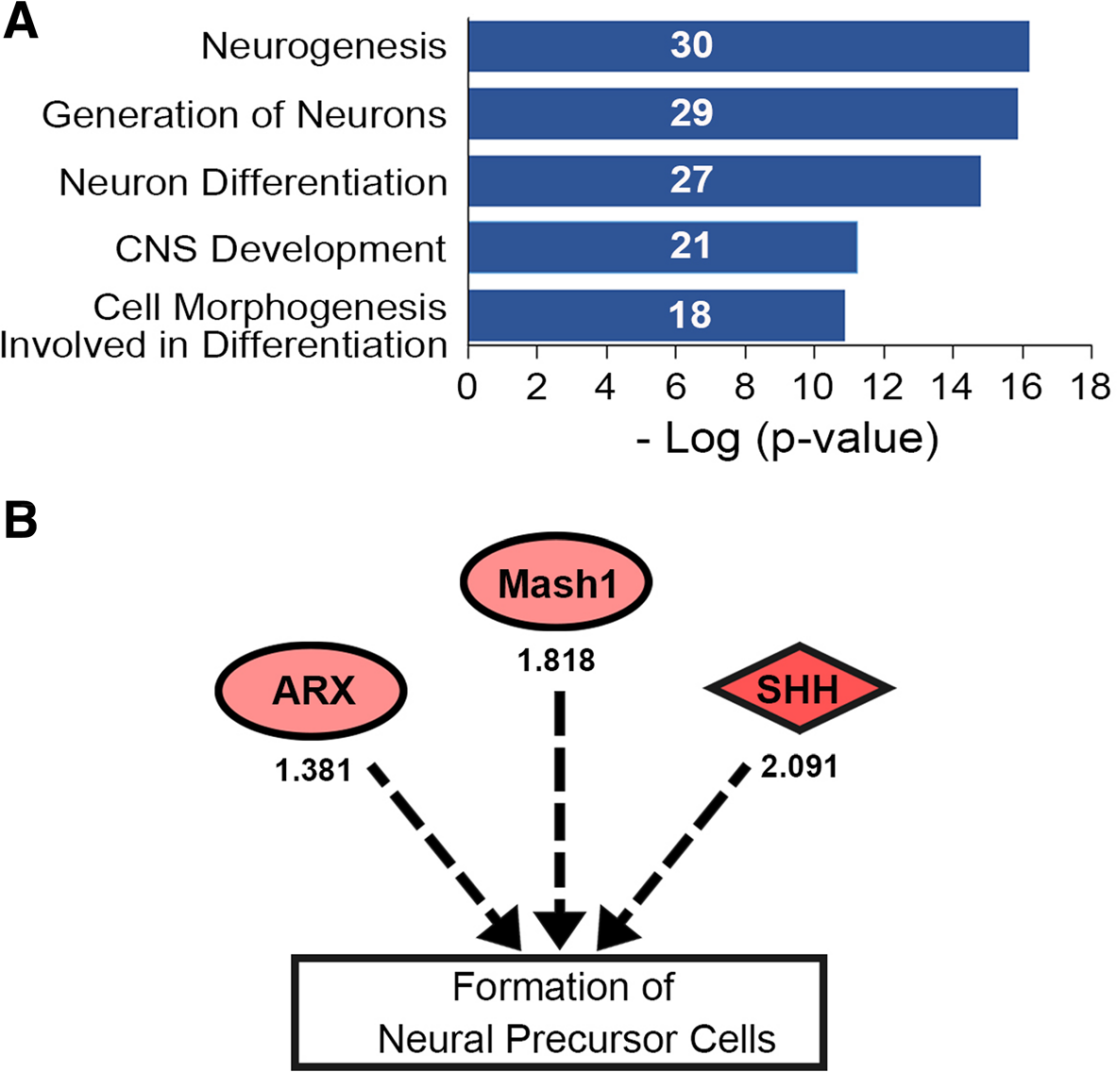
Differentially expressed genes in the embryonic brain of *Plzf*^lu/lu^ mice with functions related to neurogenesis. **a**, E10.5 forebrain and midbrain were collected for gene expression profiling by microarray. The 64 candidate genes with > or < 1.3-fold changes in expression by *Plzf*^lu/lu^ mice were further analyzed by ToppFun (https://toppgene.cchmc.org/). Using the default parameters to annotate target genes for GO Biological Process, the top five physiological system development and function categories were identified. Significance referred to the –log (*p*-value). The numbers in blue bars represent the numbers of genes involved in the category. **b**, Potential genes involved in formation of neural precursor cells. *Arx*: Aristaless related homeobox; *Ascl1 (Mash1)*: Achaete-scute family bHLH transcription factor 1; *Shh*: Sonic hedgehog. The numbers under the circles represent the fold changed between *Plzf*^lu/lu^ to *Plzf*^wt/wt^

**Fig. 6:**
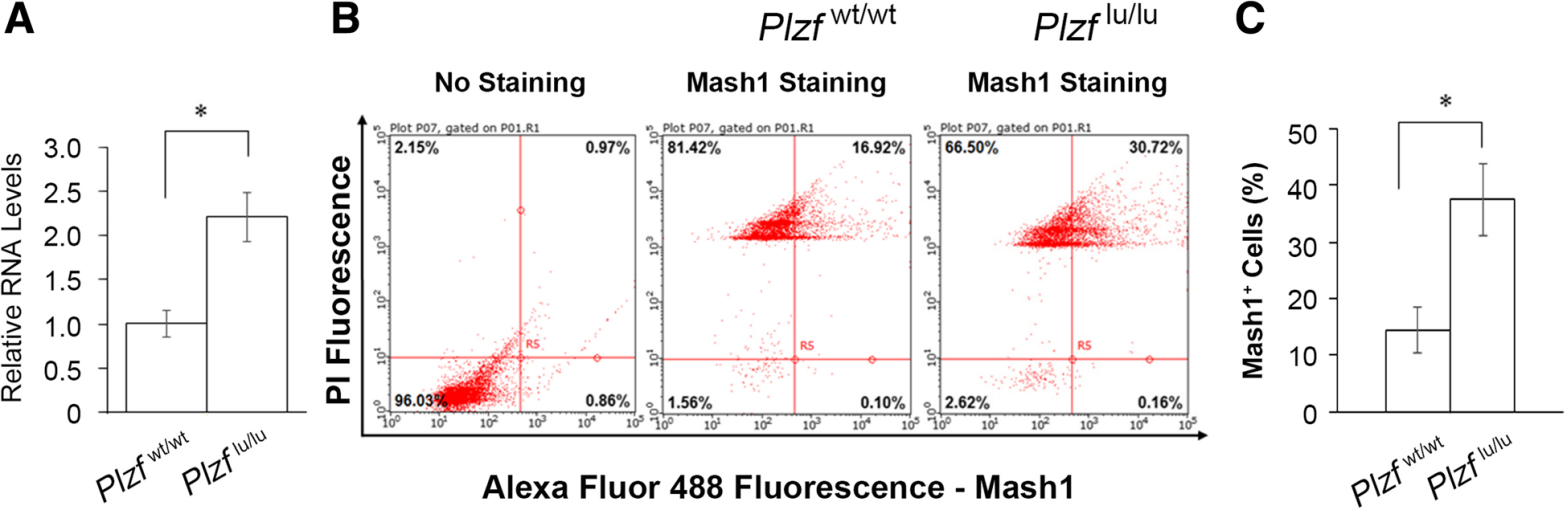
The increase of *Mash1* expression and Mash1^+^ cell population in *Plzf*^lu/lu^ mice at E10.5. **a,** qRT-PCR analysis of *Mash1* mRNA levels, normalized to GAPDH, from *Plzf*^wt/wt^ and *Plzf*^lu/lu^ mice. *Plzf*^wt/wt^, *n* = 3; *Plzf*^lu/lu^, *n* = 4. **b**, Flow cytometry dot plot shows the levels of Mash1^+^ cells in E10.5 *Plzf*^wt/wt^ and *Plzf*^lu/lu^ mice brain. Numbers at upper-right quadrant indicate the proportions of Mash1^+^ cells. **c**, Quantification of the proportions of Mash1^+^ cells. n = 4 per genotype. Values represent the mean ± SEM. Statistics performed with Student’s *t* test: *p < 0.05

Next, we compared the differences in gene expression profiles of forebrain between wild type and *Plzf*^lu/lu^ mice at E10.5 by microarray. Compared with the wild type mice, 42 genes were upregulated and 22 genes were downregulated more than 1.3-fold in the *Plzf*^lu/lu^ mice (Additional file 1: Table S1). Functional annotation of these genes was performed by ToppFun, a web database provides users to explore the functions of genes. The five highest-ranking processes in the category of ‘biological process’ were neurogenesis, generation of neurons, neuron differentiation, CNS development, and cell morphogenesis involved in differentiation (Fig. 5a and Additional file 2: Table S2). This result thus further supports the role of Plzf in brain development and neurogenesis. We also used the ingenuity pathway analysis (IPA) to further analyze these genes and found that the function of *ASCL1* (*MASH1*), *ARX*, and *SHH* were associated with the formation of neural precursor cells (Fig. 5b). Among these genes, the expression of Mash1 was related to the numbers of early-born neurons [18, 19]. Therefore, we used quantitative RT-PCR to analyze the *Mash1* RNA expression. Similar to the microarray data, the quantitative RT-PCR analysis demonstrated a significant increase in *Mash1* mRNA in *Plzf*^lu/lu^ mice when compared with wild type littermates at E10.5 (Fig. 6a). We further analyzed the cell populations by flow cytometry from the forebrain of E10.5 embryos. Our results showed a significant increase of Mash1^+^ cells in the embryos of *Plzf*^lu/lu^ mice (Fig. 6b,c), suggesting that loss of Plzf causes the early expression of Mash1.

### *Plzf*-deficient mice show a deficit in recognition memory

**Fig. 7:**
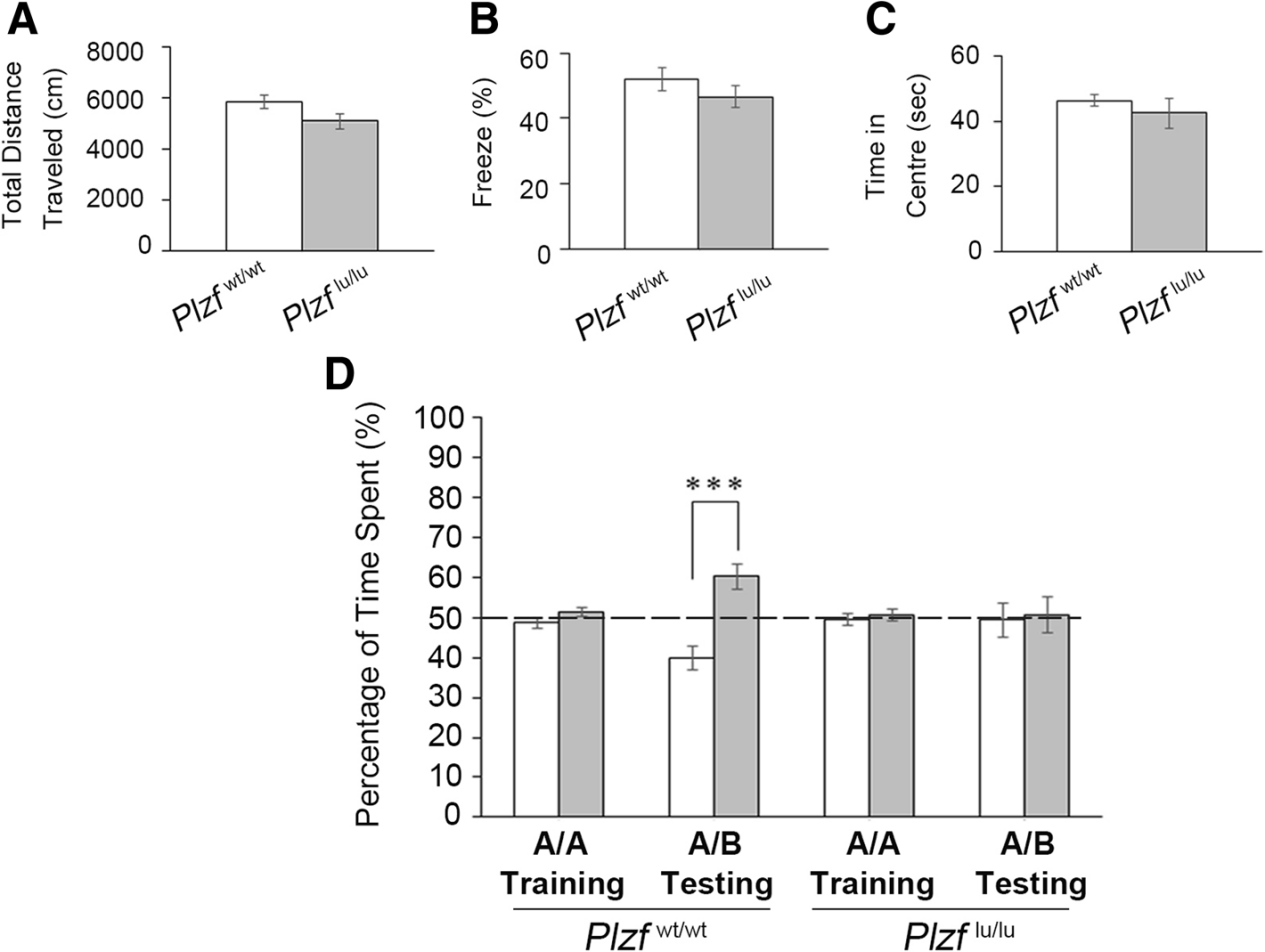
Impairment of novel object recognition memory in *Plzf*^lu/lu^ mice. **A-C,** Open field test results of *Plzf*^lu/lu^ and *Plzf*^wt/wt^ mice. **a,** Quantification of the total distance traveled; *Plzf*^wt/wt^, *n* = 16; *Plzf*^lu/lu^, *n* = 7. **b**, Quantification of the percentage of freezing time (the duration with which the mouse was completely stationary); *Plzf*^wt/wt^, *n* = 12; *Plzf*^lu/lu^, *n* = 5. **c**, Quantification of the time spent in the center zone; *Plzf*^wt/wt^, n = 16; *Plzf*^lu/lu^, n = 7. **d**, Novel object recognition test results of *Plzf*^lu/lu^ and *Plzf*^wt/wt^ mice. The % of total time the mice spent exploring each object during the training and testing sessions was recorded. The dashed line indicates 50% exploration time if none of the objects is preferred. *Plzf*^wt/wt^, *n* = 16; *Plzf*^lu/lu^, *n* = 7. Values represent the mean ± SEM. Statistical analysis was performed using Student’s t-test: *** *p* < 0.001

To further analyze the consequence of Plzf loss postnatally, we examined the functional defects in *Plzf*-deficient mice. We first performed open-field tests to assess anxiety behavior and measure basal activity. Our results showed that, although *Plzf*^lu/lu^ mice exhibited skeletal abnormalities [20], they had similar locomotor activity levels as the wild-type littermates (Fig. 7a). We also found that the duration of freezing and staying in the central zone of open field were not significantly different when compared wild-type with *Plzf*-deficient mice, suggesting that the *Plzf*-deficient mice did not exhibit more anxiety-associated behavior than wild-type littermates (Fig. 7b,c). We then analyzed the recognition memory function in wild type and *Plzf*-deficient mice using the novel object recognition test. *Plzf*^wt/wt^ and *Plzf*^lu/lu^ both explored two identical objects “A” equally during the training session (Fig. 7d). After 24 h, we placed mice with one familiar object “A” and one novel object “B” and found that wild-type mice spent significantly more time exploring the novel object “B”, suggesting that they were able to remember and recognize the familiar object (Fig. 7d). On the other hand, *Plzf*^lu/lu^ mice displayed no preference for the displaced object as opposed to the wild-type littermates (Fig. 7d). Our results thus suggest that loss of Plzf results in structural and functional abnormalities of the brain in mice.

## Discussion

The function of Plzf in brain development is less known as compared to the studies of Plzf in other biological processes such as hematopoiesis, osteogenesis, and spermatogenesis. This study demonstrated that Plzf expression was strictly regulated during brain development. Loss of Plzf resulted in microcephaly with thinner cortex and reduced numbers of Tbr1^+^ neurons in layer VI but not in other layers. PHH3 immunostaining revealed a significant decrease of mitotic cell number in *Plzf*^lu/lu^ mice at early stage of neurogenesis. Microarray, qRT-PCR, and flow cytometry analysis identified *Mash1* expression was up-regulated in the embryonic brain of *Plzf*-deficient mice at E10.5. Impairment of recognition memory was also observed in *Plzf*-deficient mice. Together, these findings clarify the effects of Plzf on the formation and function of the brain.

Plzf has been proposed to mediate a lot of essential biological processes and the *Plzf*-deficient mice, including both *Plzf* knockout and mutant mice, have been characterized in these contexts [2–4, 20, 21]. Although these studies have demonstrated defects of testis and limb homeosis and patterning in *Plzf*-deficient mice, whether the brain is also affected has not been investigated in these mouse models. Our results showing decreased cerebral cortex size and deficits in recognition memory in the *Plzf*-mutant mice (Figs. 2, 3 and 7d) are consistent with previous reports in a patient, who was identified with biallelic loss of function of the *PLZF* gene [6, 7]. In these reports, the patient was diagnosed with severe skeletal defects and genital hypoplasia. In addition, his head circumference (33 cm) was 2.4 S.D. below the mean size at birth, and the patient had severe mental retardation.

A previous study has identified an extremely dynamic expression pattern of *Plzf* mRNA during CNS development by in situ hybridization [8]. In that study, *Plzf* mRNA expression can be first detected at E7.5 in the anterior neuroepithelium and extended to the entire neuroectoderm until stage E10. The study also revealed that although Plzf was still expressed abundantly in CNS at E10.5, the expression was no longer detectable in many regions of the forebrain at E12.5. In contrast, Plzf expression in hindbrain and spinal cord remains until at least E16.5. Consistent with the result of in situ hybridization, our Western blotting results showed that Plzf protein was highly expressed in the mouse embryonic brain at E10.5 (Fig. 1a). The expression was then decreased and maintained at lower levels until E18.5. Using immunostaining, we also confirmed the dynamic expression pattern of Plzf protein in forebrain. Similar to the mRNA expression, we showed that the protein expression of Plzf was highly abundant in the forebrain at E10.5 but dramatically decreased at E11.5 (Fig. 1b-d).

We found that Plzf was expressed in the neuroepithelium before the time when neurogenesis starts (Fig. 1d), implicating that the effect of Plzf on neurogenesis may be more important in early stage than in late stage. Consistent with our speculation, we found that a significant decrease of mitotic cell number in *Plzf*^lu/lu^ mice at E10.5 (Fig. 4c) but not at E12.5 (Fig. 4d). In the spinal cord of chick and mouse, Plzf is able to promote neural progenitors proliferation and decrease neuronal differentiation [10]. In zebrafish, Plzf can block neuronal differentiation by inhibiting the expression of proneural gene Ngn1 [9]. Our result further reveals the effect of Plzf in the proliferation of neural stem cells in the brain. Loss of Plzf may result in a reduction of the neural stem/progenitor cell pool and lead to reduce the size of cerebral cortex. In mammalian brain, cerebral cortex is a highly organized, six-layered structure [22]. Among these layers, layer VI is the deepest one and the neurons in layer VI are born as early as E11.5 in mouse [23]. Two models are used to explain the mechanisms that establish cellular diversity in the cortex [24]. One is progressive restriction model, in which a single kind of radial glia cells sequentially generates all different subtypes of projection neurons and astrocytes in a defined temporal order. Base on this model, a reduction of cell proliferation in early neural progenitors should affect the number of progenitor cells in later embryogenesis, because it might reduce total number of progenitor pools. The other is lineage restriction model, in which distinct subtypes of radial glia cells co-exist and are pre-specified to generate different subtypes of projection neurons and astrocytes. Base on this model, early and later-born neurons are derived from distinct progenitor pools. We found that loss of Plzf resulted in a smaller cerebral cortex with a specific decrease in the number of neurons in layer VI but not in other layers (Fig. 3), suggesting Plzf is required for the early progenitor pools. Our results are thus in agreement with the lineage restriction model.

Our result showed an impairment of recognition memory in *Plzf*-deficient mice (Fig. 7d). Previous studies reveal that prefrontal cortex supports the hippocampus for both long-tern and short-term memory [25, 26]. And, recognition memory, in general, depends on interactions within a circuit involving the insular cortex, perirhineal cortex, ventromedial prefrontal cortex, and hippocampus [26–29]. More importantly, layer VI of visual cortex area is specifically implicated in object recognition memory formation [30]. Therefore, the impairment of recognition memory in *Plzf*-deficient mice may relate to the decrease in cerebral cortex size and the number of deep-layer cortical neurons.

Brain development is a complicated process involving neural stem cell proliferation, differentiation, and survival. Human autosomal recessive primary microcephaly (MCPH) is a congenital brain disorder caused by mutations in at least 12 different genes [31]. Patients with MCPH exhibit marked reduction in brain size and intellectual disability. To date, most of MCPH genes identified are centrosomal proteins involved in cell cycle regulation. For example, CDK5RAP2 is required to maintain centriole engagement and cohesion [32], WDR62 is related to mitotis spindle assembly and stability [33], CENPJ and PLK4 are involved in centrosome biogenesis [34, 35], and ASPM is related to spindle regulation [36]. In animal models, deficiency of MCPH genes causes brain size reduction as observed in patients [33, 37–40]. However, our data showed no change of expressions of MCPH genes in the embryonic brain of *Plzf*-deficient mice at E10.5 (Additional file 1: Table S1), implying that the effect of Plzf in cerebral cortex formation is not related to MCPH genes dysregulation.

Impaired or premature neurogenesis also affects nervous system development and causes brain size change. For example, RP58 is highly expressed in differentiating neurons to repress the expressions of proneural genes and essential for neuronal differentiation from progenitors. Loss of RP58 results in impaired neurogenesis and smaller brain size [41]. Our previous studies also reveal that Rnf112/Znf179 expresses in differentiating neurons to modulate cell cycle exit, and loss of Rnf112/Znf179 also causes smaller brain size [42, 43]. In addition to impaired neurogenesis, premature neurogenesis caused by up-regulation of proneural genes in NSCs leads to depletion of progenitor pool and reduction of brain size [44, 45]. Our result showed up-regulation of proneural gene *Mash1* expression and increase of Mash1^+^ cells in the embryonic brain of *Plzf*-deficient mice at E10.5 (Fig. 6). *Mash1*, also known as *Ascl1*, is a proneural gene regulating neurogenesis in the ventral telencephalon and is also critical for laminar fate determination of cortical neurons [18, 19, 46, 47]. As shown in the study of Hatakeyama et al., *Hes* genes including *Hes1, Hes3, and Hes5* are expressed in NSCs to repress the expressions of proneural genes such as Mash1 and contribute to the maintenance of NSCs [44]. In the absence of *Hes* genes, proneural genes (including *Mash1*) are up-regulated, leading to premature neurogenesis and concomitant a wide range of defects in brain formation. Therefore, the Mash1 dysregulation may be related to the brain malformation in *Plzf*-deficient mice.

In addition to *Mash1*, *Shh* and *Arx* are also suggested to mediate the generation of neural precursor cells and are up-regulated in the embryonic brain of *Plzf*-deficient mice at E10.5 (Fig. 5b, Additional file 1: Table S1). *Shh* gene encodes a secreted protein, sonic hedgehog, and is required for expansion of neuron precursors in cerebellum and also in neocortex [48, 49]. *Arx* is a homeobox-containing gene and regulates cell expansion of cortical intermediate progenitor [50]. Whether the *Shh* and *Arx* genes dysregulation also contributes to the defect of cerebral cortex formation in *Plzf*-deficient mice will be further examined.

## Conclusions

In conclusion, Plzf is expressed at early stages of brain development and required for the early progenitor pools. Loss of Plzf results in dysregulation of Mash1, microcephaly with reduced numbers of early-born neurons, and impairment of recognition memory.

## Additional files

Additional file 1: **Table S1.** Gene expression changes identified from a microarray of E10.5 *Plzf*^wt/wt^ and *Plzf*^lu/lu^ mice forebrain and midbrain. (DOCX 20 kb)

Additional file 2: **Table S2.** List of genes associated with the GO term. (DOCX 19 kb)

## Abbreviations

E: embryonic day
IPA: ingenuity pathway analysis
*lu*: luxoid
NOR: Novel Object Recognition
NSCs: neural stem cells
PHH3: phospho-histone H3
Plzf: promyelocytic leukemia zinc finger
