# Supplementary material for: Promyelocytic leukemia zinc finger is involved in the formation of deep layer cortical neurons
Source: J Biomed Sci. 2019 Apr 26;26:30. doi: 10.1186/s12929-019-0519-8 (PMC6485146; doi:10.1186/s12929-019-0519-8)
Supplement: Supplementary file 1 — Table S1. Gene expression changes identified from a microarray of E10.5 Plzfwt/wt and Plzflu/lu mice forebrain and midbrain. (DOCX 20 kb) [file 12929_2019_519_MOESM1_ESM.docx]

**Table S1** Gene expression changes identified from a microarray of E10.5 *Plzf*^wt/wt^ and *Plzf*^lu/lu^ mice forebrain and midbrain.

| Accession Number | Gene Symbol | *Plzf*^wt/wt^ vs. *Plzf*^lu/lu^ | | Accession Number | Gene Symbol | *Plzf*^wt/wt^ vs. *Plzf*^lu/lu^ | |
| --- | --- | --- | --- | --- | --- | --- | --- |
|  |  | **N1** | **N2** |  |  | **N1** | **N2** |
| NM_007492  NM_008553  NM_010740  NM_001081275  XM_006535725  NM_018827  NM_016669  NM_010054  NM_016885  NM_010134  NM_001290753  NM_007960  NM_010181  NM_028263  NM_001291067  NM_001160112  NM_175284  NM_008237  NM_019564  NM_010494  NM_016851  NM_010572  NM_001161536  NM_177715  NM_001097621  NM_001039000  NM_008449  NM_057173  NM_001081088  NM_028977  NM_176920  NM_145515 | Arx  Mash1  Cd93  Cfap126  Cnpy1  Crlf1  Crym  Dlx2  Emcn  En2  Ephb2  Etv1  Fbn2  Fgfbp3  Foxa2  Foxg1  Fzd10  Hes3  Htra1  Icam2  Irf6  Irs4  Islr2  Kctd12  Kif26a  Kif5a  Kif5c  Lmo1  Lrp2  Lrrc17  Lrtm1  Mark1 | 1.38  1.82  0.70  0.70  0.41  2.50  1.55  1.42  0.65  0.28  1.34  2.14  0.73  1.38  1.64  1.52  0.66  0.43  1.96  0.71  1.49  1.87  2.10  0.72  1.41  1.86  1.33  1.44  2.12  0.66  1.38  1.41 | 1.81  1.99  0.50  0.76  0.47  2.65  2.29  1.33  0.62  0.42  1.43  1.74  0.71  2.01  2.33  3.27  0.57  0.31  1.50  0.67  1.46  1.72  2.74  0.62  1.52  2.18  1.32  1.93  1.55  0.71  1.63  1.33 | NM_008665  NM_152229  NM_176930  NM_130885  NM_008781  NM_025557  NM_013892  NM_008808  NM_019932  NM_032398  NM_172775  NM_026376  NM_011141  NM_008973  NM_011218  NM_177740  AK083030  NM_017400  NM_009170  NM_011380  NR_038082  NM_011443  NR_015580  AK153771  NM_010097  NM_009133  NM_019675  NM_008536  NM_009426  NM_023716  NM_013703  NM_001033324 | Myt1  Nr2e1  Nrcam  Oxr1  Pax3  Pcp4l1  Pcsk1n  Pdgfa  Pf4  Plvap  Plxnb1  Plxnd1  Pou3f1  Ptn  Ptprs  Rgma  Rmst  Sh3gl3  Shh  Six2  Six3os1  Sox2  Sox2ot  Sox4  Sparcl1  Stmn3  Stmn4  Tm4sf1  Trh  Tubb2b  Vldlr  Zbtb16 | 1.51  1.38  2.35  1.60  0.76  0.37  1.31  2.06  0.40  0.67  1.32  0.73  1.57  0.66  1.33  1.35  0.75  1.52  2.09  1.37  1.81  1.36  1.68  1.45  0.57  1.32  1.44  0.53  0.75  1.55  1.58  0.40 | 2.60  1.92  2.22  1.65  0.62  0.48  1.43  1.56  0.74  0.58  1.31  0.55  1.72  0.57  1.37  1.76  0.73  1.47  2.05  1.50  1.35  1.39  2.77  1.54  0.66  2.18  1.91  0.71  0.64  1.65  1.89  0.39 |

Summarized data from microarray analysis. N1 and N2, two independent groups, represent the corresponding sample sizes.
