# Supplementary material for: Promyelocytic leukemia zinc finger is involved in the formation of deep layer cortical neurons
Source: J Biomed Sci. 2019 Apr 26;26:30. doi: 10.1186/s12929-019-0519-8 (PMC6485146; doi:10.1186/s12929-019-0519-8)
Supplement: Supplementary file 2 — Table S2. List of genes associated with the GO term. (DOCX 19 kb) [file 12929_2019_519_MOESM2_ESM.docx]

**Table S2** List of genes associated with the GO term.

| **Neurogenesis; GO：0022008** | | | |
| --- | --- | --- | --- |
| **Entrez Gene ID** | **Gene Symbol** | **Entrez Gene ID** | **Gene Symbol** |
| 2048 | Ephb2 | 2115 | Etv1 |
| 6657 | Sox2 | 6469 | Shh |
| 56963 | Rgma | 11211 | Fzd10 |
| 6659 | Sox4 | 5453 | Pou3f1 |
| 5764 | Ptn | 347733 | Tubb2b |
| 57611 | Islr2 | 1746 | Dlx2 |
| 7436 | Vldlr | 390992 | Hes3 |
| 81551 | Stmn4 | 5077 | Pax3 |
| 4897 | Nrcam | 3798 | Kif5a |
| 26153 | Kif26a | 3800 | Kif5c |
| 4139 | Mark1 | 23129 | Plxnd1 |
| 429 | Ascl1 | 3170 | Foxa2 |
| 50861 | Stmn3 | 2020 | En2 |
| 170302 | Arx | 2290 | Foxg1 |
| 7101 | Nr2e1 | 5364 | Plxnb1 |
| **Generation of Neurons; GO：0048699** | | | |
| **Entrez Gene ID** | **Gene Symbol** | **Entrez Gene ID** | **Gene Symbol** |
| 2048 | Ephb2 | 2115 | Etv1 |
| 6657 | Sox2 | 6469 | Shh |
| 56963 | Rgma | 11211 | Fzd10 |
| 6659 | Sox4 | 347733 | Tubb2b |
| 5764 | Ptn | 1746 | Dlx2 |
| 57611 | Islr2 | 390992 | Hes3 |
| 7436 | Vldlr | 5077 | Pax3 |
| 81551 | Stmn4 | 3798 | Kif5a |
| 4897 | Nrcam | 3800 | Kif5c |
| 26153 | Kif26a | 23129 | Plxnd1 |
| 4139 | Mark1 | 3170 | Foxa2 |
| 429 | Ascl1 | 2020 | En2 |
| 50861 | Stmn3 | 2290 | Foxg1 |
| 170302 | Arx | 5364 | Plxnb1 |
| 7101 | Nr2e1 |  |  |
| **Neuron Differentiation; GO：0030182** | | | |
| **Entrez Gene ID** | **Gene Symbol** | **Entrez Gene ID** | **Gene Symbol** |
| 2048 | Ephb2 | 2115 | Etv1 |
| 6657 | Sox2 | 6469 | Shh |
| 56963 | Rgma | 11211 | Fzd10 |
| 6659 | Sox4 | 1746 | Dlx2 |
| 5764 | Ptn | 390992 | Hes3 |
| 57611 | Islr2 | 5077 | Pax3 |
| 7436 | Vldlr | 3798 | Kif5a |
| 81551 | Stmn4 | 3800 | Kif5c |
| 4897 | Nrcam | 23129 | Plxnd1 |
| 26153 | Kif26a | 3170 | Foxa2 |
| 429 | Ascl1 | 2020 | En2 |
| 50861 | Stmn3 | 2290 | Foxg1 |
| 170302 | Arx | 5364 | Plxnb1 |
| 7101 | Nr2e1 |  |  |
| **CNS Development; GO：0007417** | | | |
| **Entrez Gene ID** | **Gene Symbol** | **Entrez Gene ID** | **Gene Symbol** |
| 2048 | Ephb2 | 7101 | Nr2e1 |
| 6657 | Sox2 | 4036 | Lrp2 |
| 6659 | Sox4 | 6469 | Shh |
| 5764 | Ptn | 5453 | Pou3f1 |
| 7436 | Vldlr | 1746 | Dlx2 |
| 7704 | Zbtb16 | 390992 | Hes3 |
| 4897 | Nrcam | 5077 | Pax3 |
| 5802 | Ptprs | 3170 | Foxa2 |
| 429 | Ascl1 | 2020 | En2 |
| 6457 | Sh3gl3 | 2290 | Foxg1 |
| 170302 | Arx |  |  |
| **Cell Morphogenesis Involved in Differentiation; GO：0000904** | | | |
| **Entrez Gene ID** | **Gene Symbol** | **Entrez Gene ID** | **Gene Symbol** |
| 2048 | Ephb2 | 6469 | Shh |
| 56963 | Rgma | 5077 | Pax3 |
| 57611 | Islr2 | 3798 | Kif5a |
| 7436 | Vldlr | 3800 | Kif5c |
| 4897 | Nrcam | 23129 | Plxnd1 |
| 26153 | Kif26a | 3170 | Foxa2 |
| 170302 | Arx | 10736 | Six2 |
| 7101 | Nr2e1 | 2290 | Foxg1 |
| 2115 | Etv1 | 5364 | Plxnb1 |

GO, gene ontology; CNS, central nervous system.
